# Immune modulatory roles of radioimmunotherapy: biological principles and clinical prospects

**DOI:** 10.3389/fimmu.2024.1357101

**Published:** 2024-02-21

**Authors:** Xuefeng Wang, Yu Wang, Yonggang Zhang, Hongyun Shi, Kuan Liu, Fang Wang, Yue Wang, Huijing Chen, Yan Shi, Ruiyao Wang

**Affiliations:** ^1^ Department of Radiation Oncology, Affiliated Hospital of Hebei University, Baoding, Hebei, China; ^2^ Department of Radiation Oncology, National Cancer Center/National Clinical Research Center for Cancer/Cancer Hospital, Chinese Academy of Medical Sciences and Peking Union Medical College, Beijing, China; ^3^ Department of Head and Neck Surgery, Affiliated Hospital of Hebei University, Baoding, Hebei, China; ^4^ Department of Medical Oncology, Affiliated Hospital of Hebei University, Baoding, Hebei, China; ^5^ Department of Thoracic Surgery, Affiliated Hospital of Hebei University, Baoding, Hebei, China

**Keywords:** radiotherapy, immune modulation, immune checkpoint inhibitors, abscopal effect, review

## Abstract

Radiation therapy (RT) not only can directly kill tumor cells by causing DNA double-strand break, but also exerts anti-tumor effects through modulating local and systemic immune responses. The immunomodulatory effects of RT are generally considered as a double-edged sword. On the one hand, RT effectively enhances the immunogenicity of tumor cells, triggers type I interferon response, induces immunogenic cell death to activate immune cell function, increases the release of proinflammatory factors, and reshapes the tumor immune microenvironment, thereby positively promoting anti-tumor immune responses. On the other hand, RT stimulates tumor cells to express immunosuppressive cytokines, upregulates the function of inhibitory immune cells, leads to lymphocytopenia and depletion of immune effector cells, and thus negatively suppresses immune responses. Nonetheless, it is notable that RT has promising abscopal effects and may achieve potent synergistic effects, especially when combined with immunotherapy in the daily clinical practice. This systematic review will provide a comprehensive profile of the latest research progress with respect to the immunomodulatory effects of RT, as well as the abscopal effect of radioimmunotherapy combinations, from the perspective of biological basis and clinical practice.

## Introduction

Cancer remains the leading disease burden worldwide (1–3). Radiation therapy (RT) plays an important role in the treatment of cancers and is an effective local treatment method. Traditionally, it is wide acknowledged that RT leads to DNA double stand breaks (DSBs) and thereby kills tumor cells (4). In recent years, multiple studies have suggested that RT could exert anti-tumor immune effects by regulating local and systemic immune responses (5). Currently, with the development of immune checkpoint inhibitors (ICIs), the immune modulatory effect of RT and the synergistic effect of radioimmunotherapy combinations have attracted extensive attention and discussions (6, 7). However, the immune modulatory effect of RT has a double-sided nature: it can enhance the host’s anti-tumor immune response, but it may also produce immune suppression effects under certain conditions (8). The key molecular mechanisms of RT promoting or inhibiting adaptive and innate anti-tumor immune responses not only have triggered numerous exploration and investigations, but also remain the research hotspot now and in the future (9).

In addition, in the clinical practice of combining RT with ICI treatments, it has been observed that effective anti-tumor immune responses can occur at distant lesions outside the irradiation field, known as the “abscopal effect”, further emphasizing the immune modulatory and synergistic effects of RT (10–13). Therefore, the combinatorial use of RT and ICIs may produce complex interactions. This review focuses on the latest research progress on the immune modulatory effects of RT and systematically summarizes the theoretical basis and clinical evidence for the synergistic effects of radioimmunotherapy, aiming to elucidate the biological mechanisms and practical principles when combining RT with ICIs and provide reference for improving the comprehensive cancer treatment.

## Immune-activating effect of radiation therapy

### Induce immunogenic cell death to promote T cell immune response

The key molecular mechanism that ionizing radiation promotes anti-tumor immune responses is mainly by inducing the immunogenic cell death (ICD), which leads to the release of specific antigens from tumor cells and the stimulation of clone expansion in tumor-specific T lymphocyte subsets (14, 15). Antigen-presenting cells (APCs) capture specific antigens and present them in conjunction with major histocompatibility complex (MHC) to activate helper T cells (Th), which can include cytotoxic T lymphocytes (CTLs) and natural killer (NK) cells to exert anti-tumor immune effects and eliminate tumor cells (16, 17). Overall, ICD induced by RT can effectively stimulate T lymphocyte recruitment and differentiation to recognize and kill tumor cells (18, 19).

Prior studies have suggested that RT can induce oxidative stress sources, such as reactive oxygen species (ROS), leading to endoplasmic reticulum stress responses and mediating ICD (20, 21). This process is accompanied by an increase in antigen release and damage-associated molecular patterns (DAMPs), which participate in the activation of immune response signaling pathways and facilitate anti-tumor immune responses (22). DAMPs are one of the most crucial molecular steps during the radiation-induced ICD. DAMPs include cell surface expression of calreticulin and heat shock proteins, release of high mobility group box 1 protein, and active secretion of adenosine triphosphate (23). In addition, DAMPs can upregulate the expression of tumor-associated antigens (TAAs), that is, primarily neoantigens that are immunogenic mutations induced by ionizing radiation. With the release of inflammatory cytokines, DAMPs can also enhance the function of cytotoxic CD8^+^ T cells (15, 24). Recent research has also shown that RT can further reshape the T cell receptor repertoire of tumor-infiltrating lymphocytes (TILs) (25, 26).

### Activate cGAS-STING pathway to induce type I interferon response

Stimulator of interferon genes (STING) is an endoplasmic reticulum membrane protein that regulates innate immune signaling (27). Cyclic GMP-AMP synthase (cGAS) is a nucleotidyltransferase that senses cytoplasmic DNA and activates the STING-TBK1-IRF-3 signaling axis, thereby producing type I interferon signaling (28). The cGAS-STING pathway is crucial to innate immune responses, anti-viral immune responses, and tumor adaptive immunity (24). Another pivotal mechanism by which RT promotes anti-tumor immune effects is activating the cGAS-STING pathway, subsequently triggering type I interferon cascade reactions, and recruiting APCs to capture and cross-present TAAs to deploy cytotoxic CD8^+^ T-cell functions (24, 28). Specifically, RT promotes the release of double-stranded DNA (dsDNA) in the cell nucleus, increases the permeability of the outer mitochondrial membrane, and triggers the exposure of mitochondrial DNA (mtDNA) in the cytoplasm (29). Both dsDNA and mtDNA are effective mediators for initiating the cGAS-STING pathway and the transcription of type I interferons (30, 31). The type I interferon signal further activates dendritic cells (DCs). After being matured, DCs present antigens to T cells. Tumor antigen-specific T cell effector functions is therewith activated, the number of effector lymphocytes increases, and macrophage activity is also promoted, resulting in the amplification of adaptive anti-tumor immune responses (31).

### Enhance MHC-I expression and increase the visibility of antigen

MHC-I molecules bind to endogenous antigen peptides produced within cells and are capable of displaying and conveying antigenic information on the cell surface (32). By binding to CD8^+^ T cells, MHC-I molecules enable the recognition and effective killing of pathological cells that synthesize abnormal proteins, such as tumor cells that express mutated proteins (33, 34). MHC-I tumor antigens play an important role in anti-tumor immune responses. However, during the development of malignant tumors, tumor cells often lack or have low expression of MHC-I molecules to evade the recognition, immune surveillance, and attack by T lymphocytes (33, 34). Therefore, tumor cells could achieve immune escape by losing MHC-I antigen expression, which not only damages the anti-tumor effect of innate immune responses, but also weakens the therapeutic effect produced by some immune checkpoint inhibitors that can reactivate CD8^+^ T cells to exert anti-tumor effects (35). Many recent studies have indicated that RT can significantly increase the expression of MHC-I on the surface of tumor cells and promote the generation of TAAs (36, 37). This can expand the antigen pool that can be presented by APCs, improve the ability of CTL to recognize tumor cells, increase the visual imprint of the host immune system on tumor cells, effectively reduce tumor escape, and enhance anti-tumor immune responses (34).

### Release proinflammatory cytokines to activate tumor microenvironment

In addition to directly killing tumor cells, RT regulates tumor immune microenvironment (TIME) and transforms it from an immunosuppressive “cold” to immune-activated “hot” tumors. RT can stimulate the release of many pro-inflammatory chemokines, including CXCL9, CXCL10, CXCL11, and CXCL16, from tumor cells and stromal cells, which promote the immune infiltration and increase the cell abundance of DCs, macrophages, and T lymphocytes, thereby effectively activating TIME (38, 39). Recent research has demonstrated that conventional fractionated RT with 2 Gy per fraction could reprogram the phenotype of tumor-associated macrophages (TAMs), making them more prone to promote immune antigenicity and increase their anti-tumor immunity (40). In general, TAMs have shown to inhibit T lymphocytes and accelerate tumor metastases, whereas after polarization they could exhibit anti-tumor effects. RT can promote the polarization of M2-like macrophages towards inducible nitric oxide synthase (iNOS)-positive M1-like polarized macrophages. Though M2-like macrophages express CD206 and Arg-1 and release anti-inflammatory cytokines, M1 iNOS-positive macrophages can induce Th1 chemokine expression, release a variety of inflammatory cytokines, recruit CD8^+^ and CD4^+^ T cells, and promote T cell-mediated anti-tumor responses (41, 42). Hence, the theoretical principle of RT driving stress signals to reshape TIME mainly lies in the fact that RT can increase various immune regulatory proteins, adhesion molecules, cytokines, and pro-oxidants, positively activating TIME and anti-tumor immune responses.

### Upregulate the expression of death receptor on tumor cell surface

**Figure 1:**
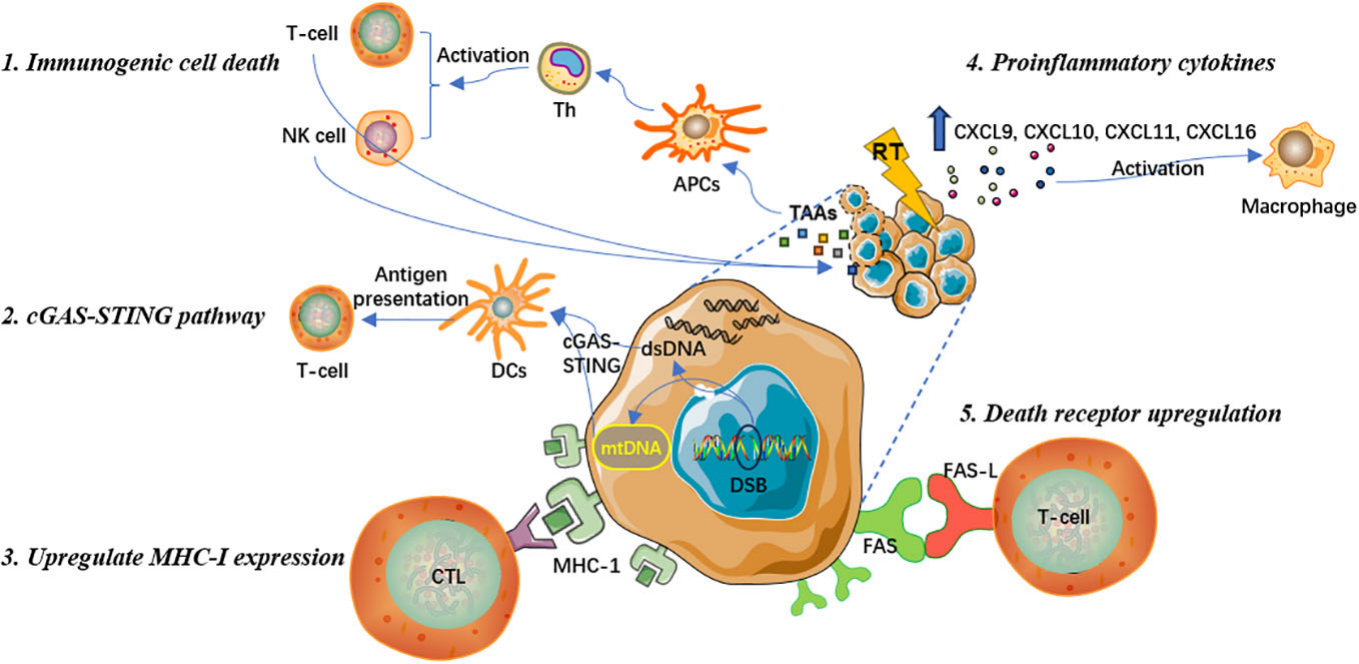
Mechanisms of the immune-activating effect of radiotherapy.

FAS, a member of the death receptor family and expressed on the cell surface, is essential to initiate programmed cell death signaling (43). The combination of FAS and its specific ligand FAS-L can enable the recruitment of the death-inducing signaling complex and proteolytic activation of effector caspases 3, 6 and 7 that mediate apoptosis, resulting in cytotoxic signals and effectively promoting the local and systemic anti-tumor immune response (43, 44). Studies have shown that RT can activate the endogenous apoptotic signaling pathway, upregulate the expression of FAS apoptotic receptors on the surface of tumor cells, mediate the effective binding of CTLs and FAS on tumor cells, and promote tumor cell apoptosis (45). Therefore, the upregulation of FAS expression is one of the critical mechanisms by which RT increases the susceptibility of tumor cells to immune response-mediated cell death (43). In conclusion, local RT can exert immune-activating effect through various ways, which has obvious advantages and wider clinical application prospect. Specific mechanisms are summarized in **Figure 1**.

## Immunosuppressive effect of radiation therapy

### Induce chronic type I interferon and interferon-stimulated gene expression

RT can cause accumulation of dsDNA in tumor cells, which activates the cGAS/STING pathway and promotes the transcription of type I interferon genes (46). STING can activate different interferon-stimulated genes through its downstream signaling pathway. However, in some cases, interferon signaling may also have negative effects. For example, repeated irradiation of tumor cells could induce chronic type I interferon and interferon-stimulated gene expression, which could make effector T cells to express more inhibitory factors and exhaust T cells, leading to treatment resistance and tumor immune escape via multiple inhibitory pathways (47). Studies have illustrated that prolonged interferon signaling was synergistically associated with programmed cell death ligand-1 (PD-L1)-dependent and programmed cell death protein-1 (PD-1)-independent ICI resistance, as well as resistance to radioimmunotherapy (48). Continued interferon signal transduction enables tumor cells to acquire signal transducer and activator of transcription 1 (STAT1)-related epigenomic changes and increase the expression of interferon-stimulated genes and various T cell inhibitory receptor ligands (48, 49). Moreover, both type I and type II interferons can induce the above mechanisms of tumor resistance to treatments.

### Upregulate expression of PD-L1 and IDO on tumor cell surface

It is generally accepted that RT could activate the cGAS-STING signaling pathway and thus promote the transcription of interferon-stimulated genes. Nevertheless, interferon-gamma and type I interferon could also upregulate the expression of PD-L1 on the surface of tumor cells, which could increase the immune escape of tumor cells and further induce T lymphocyte exhaustion, weakening the anti-tumor immune response (50). In addition, research indicated that RT not only upregulated the expression of PD-L1 on tumor cells, but also could regulate the expression of multiple immune checkpoint ligands on the surface of immune cells in the tumor microenvironment, producing suppressive tumor immune effects (51, 52). Furthermore, indoleamine 2,3-dioxygenase (IDO), a crucial enzyme involved in the tumor proliferation and immune suppression, could be upregulated by interferon-gamma and type I interferon as an immune inhibitory factor (53–55). Previous studies demonstrated that IDO could result in T cell exhaustion and further upregulate the expression of inhibitory receptors and ligands (55). Meanwhile, the overexpression of IDO on the surface of DCs was associated with decreased T lymphocyte proliferation and poor clinical prognosis in multiple cancer types (55, 56).

### Promote and enhance the function of inhibitory immune cells

The STING signaling pathway activated by RT can further enhance the recruitment of regulatory T cells (Tregs) and facilitate the development of myeloid-derived suppressor cells (MDSCs), consequently eliminating the tumor immunogenicity, counteracting the immunostimulatory properties of radiation, and causing immunosuppression (24, 52, 57). Both Tregs and MDSCs exert immunosuppressive effects in immunological responses to cancers and other diseases through various pathways and mechanisms (57, 58). MDSCs express Arg-1 and iNOS, produce ROS, and downregulate anti-tumor immune activity via the release of different chemicals and factors *in vivo* (59–61). Local irradiation of tumor lesions could increase the production of chemokine ligand (CCL)2 and CCL5, which are associated with the recruitment of Tregs and monocytes (62, 63). Recruited monocytes activate Tregs through the tumor necrosis factor-alpha (TNF-α) mediated pathway, which suppresses anti-tumor immune responses and further reduces therapeutic efficacy (64). Besides, by secreting interleukin-10 (IL-10), transforming growth factor-beta (TGF-β), and other cytokines, Tregs can not only enhance the immunosuppressive function of MDSCs, but also inhibit the immune function of effector T cells (65–68).

### Cause lymphopenia and depletion of immune effector cells

**Figure 2:**
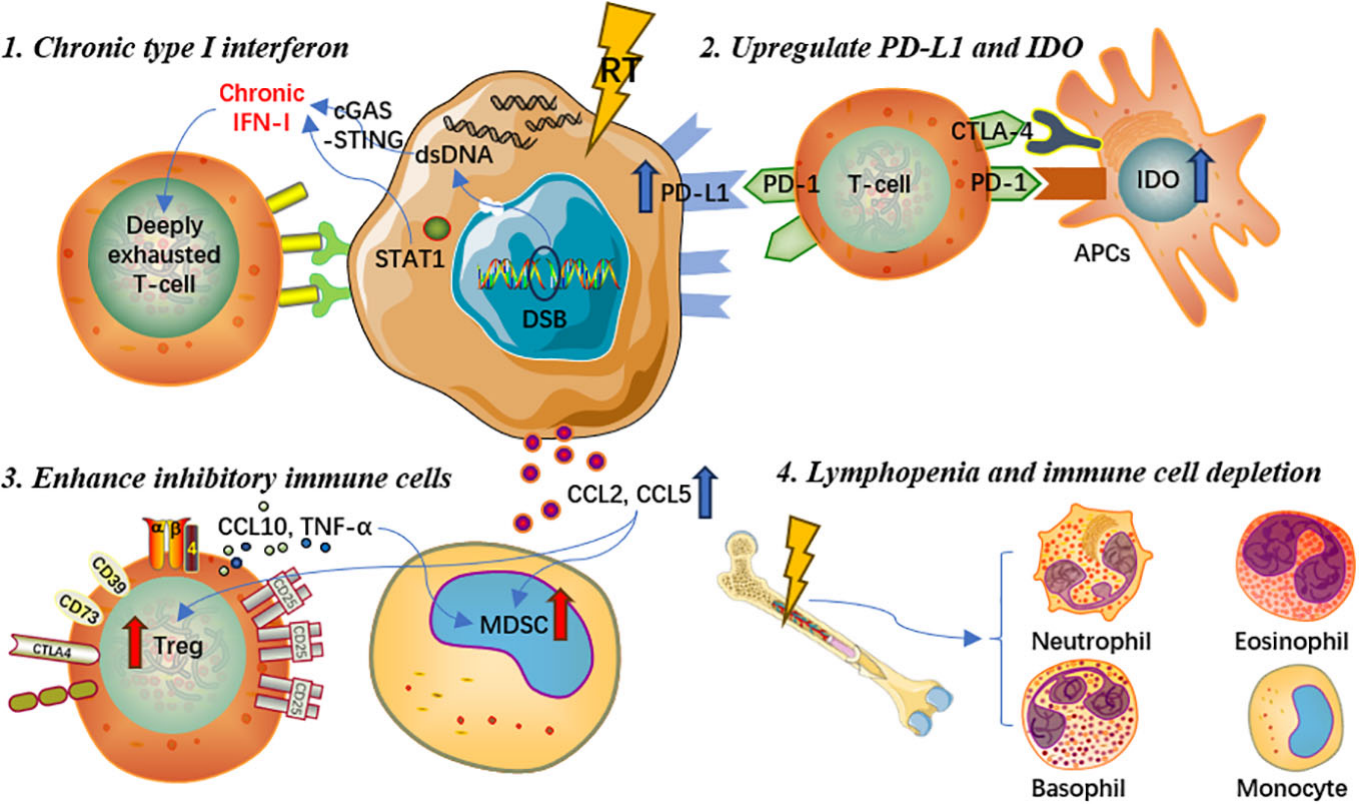
Mechanisms of the immunosuppressive effect of radiotherapy.

Lymphopenia is one of the most common adverse events during and after RT in a daily basis, and is deemed to be associated with poorer survival prognosis for cancer patients (69, 70). Given that hematopoietic stem cells are sensitive to ionizing radiation, even low-dose irradiation may cause temporary bone marrow dysfunction, while high-dose RT may result in irreversible damage to bone marrow hematopoietic function and mesenchymal stromal cells (71–73). In real-world clinical settings, patients are often given a certain dose of irradiation which can achieve the purpose of killing tumor cells, whereas some patients could experience severe bone marrow dysfunction, resulting in a significant decrease in lymphocyte count and accordingly decreased anti-tumor immune response (74). Chen et al(75) found that lymphopenia post-RT could affect the occurrence of abscopal responses and thus negatively influence prognosis in patients treated with RT and immunotherapy. Similarly, monocytes in the peripheral blood circulation are highly sensitive to ionizing radiation. Repeated conventional fractionated RT for 5 consecutive days per week may cause potential cell toxicity damage, deplete immune effector cells that migrate to the peripheral circulation, accelerate aging-related clonal hematopoiesis, and eventually lead to immunosuppressive effects (76). Another potential mechanism for radiation-induced lymphocyte reduction is the irradiation of lymphoid organs. Due to the extreme sensitivity of immature T cells to RT, even low-dose irradiation of lymphoid organs could contribute to rapid p53-mediated apoptosis, which is related to reduced lymphocyte count, increased T cell apoptosis activity, as well as poorer prognosis (62). Hence, lymphopenia, cytotoxic effects on leukocytes, and depletion of immune effector cells are also important reasons for the immunosuppressive effects caused by RT. In brief, RT could also play a negative role in modulating the systemic immune system, which is worthy of further elaboration in future research. Detailed mechanisms of the immunosuppressive effect are presented in **Figure 2**.

## Abscopal effect of radiation combined with immunotherapy

### Clinical application and prospect of abscopal effect

About 60 years ago, radiation oncologists discovered the “abscopal effect” of RT, that is, the effective treatment response of tumor shrinkage was observed at a distant site out of the radiation field (77). Although there were merely 47 literatures regarding the abscopal effect reported between 1960 and 2018, this number has rapidly surged after the advent of immunotherapy, presumably because the combination of RT and ICIs could effectively promote anti-tumor effects of the immune system (78). In 2012, Postow et al(79) first reported the abscopal effect of RT in combination with immunotherapy in a case report: a patient with melanoma who received local RT on oligometastatic sits and ipilimumab, a cytotoxic T lymphocyte-associated antigen-4 (CTLA-4) inhibitor, exhibited regression of distant lesions outside the radiation field. Subsequently, mounting evidence has reported the abscopal effect of combining RT with ICIs, and indicated the increased infiltration of immune cells and the enhancement of anti-tumor immune response outside the radiation field. In 2015, Golden et al(80) conducted a proof-of-principle clinical trial in which the immunogenicity of granulocyte macrophage-colony stimulating factor (GM-CSF) was regulated by irradiation, and the effect of RT was validated in clinic for the first time. This study adopted a Simon two-stage design and included a total of 41 patients. In the phase I stage with 10 subjects, abscopal effects were observed in 4 patients. In the phase II stage, 31 additional patients were included, and 11 of the cumulative 41 patients (26.8%) developed abscopal effects. Overall, this research is the first clinical evidence that the combination of RT and immunotherapy can induce the abscopal effect in solid metastatic tumors, and distant remission of metastatic sites can predict better survival outcomes (80).

In 2018, Formenti et al(81) found that in advanced non-small-cell lung cancer (NSCLC) patients with resistance to chemotherapy, RT combined with CTLA-4 inhibitors effectively induced systemic T lymphocyte anti-tumor responses. In this study, CTLA-4 inhibitor alone or in combination with chemotherapy had unsatisfactory efficacy, whereas CTLA-4 inhibitor plus RT showed significant anti-tumor effects (81). Exploratory analysis of the peripheral blood specimens from subjects indicated that the increase of serum interferon β and the early dynamic change of T cell cloning after RT were potent predictors of efficacy (81). Moreover, one patient with complete response revealed a large expansion of CD8^+^ T cells and the recognition of neoantigens encoded by genes upregulated after RT (81). Hence, the mechanisms of the abscopal effect explained in this study were as follows: After exposure to the systemic immune system of the immunogenic mutation induced by RT, tumor cells in the irradiated field were attacked by circulating immune cells and thus demonstrated distant anti-tumor responses. At present, the exact mechanism and principle of the abscopal effect of RT combined with ICIs observed in clinic remain unclear and warrant further investigations (82).

In recent year, the abscopal effect of RT in combination with immunotherapy has attracted increasing attention from the public. In the secondary analysis of the KEYNOTE-001 trial (83), patients treated with pembrolizumab and RT exhibited significantly longer progression-free survival (PFS; median 4·4 vs 2·1 months; hazard ratio [HR] 0·56; P=0·019) and overall survival (OS; median 10·7 vs 5·3 months; HR 0·58; P=0·026) than patients without previous RT. These data suggest that RT combined with pembrolizumab treatment could bring the synergistic survival benefits to patients with advanced NSCLC (83). In the randomized phase II PEMBRO-RT study (84), compared with pembrolizumab alone, stereotactic body radiotherapy (SBRT) prior to pembrolizumab brought a doubling of overall response rate (36% vs 18%; P=0·070) and a significantly prolonged PFS (median 6.6 vs 1.9 months; HR 0·58; P=0·026). Subgroup analyses further showed the largest benefit from the addition of RT in patients with PD-L1-negative tumors, implying that RT may activate non-inflamed NSCLC toward a more inflamed tumor microenvironment (84). Additionally, a pooled analysis of the PRMBRO-RT (phase II) and MDACC (phase I/II) trials demonstrated significantly improved PFS (median 9·0 vs 4·4 months; HR 0·67; P=0·045) and OS (median 19·2 vs 8·7 months; HR 0·67; P<0·001) with pembrolizumab plus RT than pembrolizumab alone in patients with metastatic NSCLC (85). Meanwhile, both the best out-of-field (abscopal) response rate (41.7% vs 19.7%; P=0·004) and best abscopal disease control rate (65.3% vs 43.4%; P=0·007) was significantly greater with pembrolizumab plus RT versus with pembrolizumab alone, highlighting the significantly increased antitumoral responses and augmented survival benefit noted in the combination treatment (85). In hepatocellular carcinoma, SBRT and ICI combinations were also found potentially effective in inducing the immunomodulatory effects as an”*in situ* vaccine” to increase T-cell receptor diversity and further result in out-of-field abscopal antitumor effects (86).

### Limitations of abscopal effect

In clinical practice, there are many factors affecting the abscopal effect of RT combined with ICIs, including radiation dose and segmentation, irradiation sites, general condition of patients, disease stage, tumor characteristics, the sequence of RT and ICIs, and the selection of different ICI agents (7, 82). While radiation can activate the immune system, the optimal dose and timing of RT for the maximal abscopal effect is not fully understood (87). In terms of the radiation dose and segmentation, prior research implied that the positive activating effects of RT on immune responses may be “dose-dependent” within a certain range, and higher single dose RT of ≥15 Gy (12-18 Gy) could lead to increased immunosuppressive effects, such as the accumulation of CD4^+^ FoxP3^+^ Treg or Trex1 induction to attenuate tumor immunogenicity (88–90). Nevertheless, other studies suggested different RT doses and segmentations played various immunomodulatory role (87). Some scholars considered low-dose RT, which is commonly used for patients with metastatic diseases as palliative care (91, 92), can better induce anti-tumor immune activation at the molecular level, reshape TIME, and improve the infiltration and function of effector immune cells in distant tumor foci (9, 93–95). Therefore, anti-tumor responses outside the radiation field strengthened by low-dose RT were termed the “RadScopal effect” by them (9, 96). Positive and negative responses of radioimmunotherapy-induced abscopal effect are summarized in **Table 1**.

**Table 1:** Clinical evidence for radioimmunotherapy-induced abscopal response.

Study	Study Type	Type of Cancer	Treatment	Abscopal Response
Postow et al(2012) (79)	Case report	Melanoma	SBRT (28.5 Gy/3 fractions/9.5 Gy) + Ipilimumab	Positive
Golden et al(2015) (80)	Proof-of-principle trial	Metastatic solid tumors	RT (35 Gy/10 fractions/3.5 Gy) + GM-CSF	Positive in 11/ 41 patients (26.8%); Negative in 73.2%
Formenti et al(2018) (81)	Two-satge phase I/II	Metastatic NSCLC	SBRT (30 Gy/5 fractions/6 Gy in phase I, 28.5 Gy/3 fractions/9.5 Gy in phase II) + Ipilimumab	Positive in 12/39 patients (31%); Negative in 69%
Shaverdian et al(2017)/KEYNOTE-001 (83)	Phase I	Metastatic NSCLC	Previous RT + Pembrolizumab	Positive (mPFS 4·4 ms, mOS 10.7 ms)
Theelen et al(2019)/PEMBRO-RT (84)	Phase II	Metastatic NSCLC	Privious SBRT (24 Gy/3 fractions/8 Gy) + Pembrolizumab	Positive (12-week ORR 36%, mPFS 6.6 ms, mOS 15.9 ms)
Theelen et al(2021) (85)	Pooled analysis of phase II (PEMBRO-RT) and phase I/II (MDACC)	Metastatic NSCLC	PEMBRO-RT: Privious SBRT (24 Gy/3 fractions/8 Gy) + Pembrolizumab MDACC: Concurrent RT (50 Gy/4 fractions/12.5 Gy or 45 Gy/15 fractions/3 Gy) + Pembrolizumab	Positive (best ARR 41.7%, best ACR 65.3%, mPFS 9.0 ms, mOS 19.2 ms)
Menon et al(2019) (95)	Post-hoc analysis of two phase I/II and one phase II	Metastatic tumors	LDRT (1-20 Gy total) + Ipilimumab or Pembrolizumab or other immunotherapy	Postive in 22/38 patients (58%); Negative in 42%

SBRT, stereotactic body radiotherapy; Gy, gray; RT, radiation therapy; GM-CSF, granulocyte macrophage-colony stimulating factor; NSCLC, non-small-cell lung cancer; mPFS, median progression-free survival; mOS, median overall survival; ms, months; ORR, overall response rate; ARR, abscopal response rate; ACR, abscopal disease control rate; LDRT, low-dose radiation therapy.nical evidence for radioimmunotherapy-induced abscopal response.

Taken together, the immunomodulatory effect of RT is two-sided. On the one hand, it can enhance anti-tumor immune effect through various mechanisms; on the other hand, it may have immunosuppressive effect in certain cases. The key principles of RT to promote local and systemic anti-tumor immune responses include: inducing ICD to facilitate T lymphocyte proliferation; activating cGAS-STING pathway to promote type I interferon response; upregulating the expression of MHC-I on the surface of tumor cells; and enhancing the immunogenicity and antigen visibility of tumor cells; stimulating the release of various proinflammatory cytokines in tumor cells and stromal cells to reshape TIME; increasing immune checkpoint and FAS expression on tumor cell surface to enhance the anti-tumor immune effect. On the contrary, the negative immunosuppressive mechanism mainly includes: RT induced chronic type I interferon and interferon-stimulated gene expression; upregulating PD-L1 and IDO expression on tumor surface; promoting the inhibitory immune cell functions; causing lymphocytopenia and depletion of immune effector cells. At the same time, the abscopal effect of RT and the radscopal effect of low-dose RT combined with ICIs, which constitute an important basis for the synergistic effect, brought substantial therapeutic benefits during the clinical practice. Currently, the best combination modality of RT plus ICIs remains uncertain and warrants further in-depth research and more exploration in the future, which is expected to significantly improve the survival prognosis of cancer patients, promote the scientific progress of comprehensive treatments, and facilitate the development of accurate cancer personalization.

## Author contributions

XW: Conceptualization, Data curation, Investigation, Methodology, Project administration, Resources, Software, Visualization, Writing – original draft. YuW: Conceptualization, Data curation, Investigation, Methodology, Project administration, Resources, Software, Visualization, Writing – original draft. YZ: Conceptualization, Data curation, Investigation, Methodology, Project administration, Resources, Software, Visualization, Writing – original draft. HS: Conceptualization, Investigation, Project administration, Writing – original draft. KL: Conceptualization, Investigation, Project administration, Writing – original draft. FW: Conceptualization, Investigation, Project administration, Writing – original draft. YueW: Conceptualization, Investigation, Project administration, Writing – original draft. HC: Conceptualization, Investigation, Project administration, Writing – original draft. YS: Conceptualization, Investigation, Project administration, Supervision, Writing – original draft, Writing – review & editing. RW: Conceptualization, Investigation, Project administration, Supervision, Writing – original draft, Writing – review & editing.
